# Does the Benefit on Survival from Leisure Time Physical Activity Depend on Physical Activity at Work? A Prospective Cohort Study

**DOI:** 10.1371/journal.pone.0054548

**Published:** 2013-01-17

**Authors:** Andreas Holtermann, Jacob Louis Marott, Finn Gyntelberg, Karen Søgaard, Poul Suadicani, Ole Steen Mortensen, Eva Prescott, Peter Schnohr

**Affiliations:** 1 The National Research Centre for the Working Environment, Copenhagen, Denmark; 2 The Copenhagen City Heart Study, Frederiksberg Hospital, Copenhagen, Denmark; 3 The Copenhagen Male Study, Epidemiological Research Unit, Department of Occupational and Environmental Medicine, Bispebjerg University Hospital, Copenhagen, Denmark; 4 Institute of Sport Science and Clinical Biomechanics, University of Southern Denmark, Odense, Denmark; 5 Department of Cardiology, Bispebjerg University Hospital, Copenhagen, Denmark; NIDDK/NIH, United States of America

## Abstract

**Purpose:**

To investigate if persons with high physical activity at work have the same benefits from leisure time physical activity as persons with sedentary work.

**Methods:**

In the Copenhagen City Heart Study, a prospective cohort of 7,411 males and 8,916 females aged 25–66 years without known cardiovascular disease at entry in 1976–78, 1981–83, 1991–94, or 2001–03, the authors analyzed with sex-stratified multivariate Cox proportional hazards regression the association between leisure time physical activity and cardiovascular and all-cause mortality among individuals with different levels of occupational physical activity.

**Results:**

During a median follow-up of 22.4 years, 4,003 individuals died from cardiovascular disease and 8,935 from all-causes. Irrespective of level of occupational physical activity, a consistently lower risk with increasing leisure time physical activity was found for both cardiovascular and all-cause mortality among both men and women. Compared to low leisure time physical activity, the survival benefit ranged from 1.5–3.6 years for moderate and 2.6–4.7 years for high leisure time physical activity among the different levels of occupational physical activity.

**Conclusion:**

Public campaigns and initiatives for increasing physical activity in the working population should target everybody, irrespective of physical activity at work.

## Introduction

A sedentary lifestyle is an established risk factor for cardiovascular disease and mortality [1]–[3]. This statement is based on considerable documentation for health benefits from physical activity on cardiovascular disease and mortality [4]–[8]. Accordingly, public campaigns, initiatives, and workplace health promotion for increasing physical activity, are often targeting persons with sedentary work [9].

Even today a considerable proportion of work active people have physically demanding jobs [10]–[12]. During the last decade, a number of studies have shown that individuals with high occupational physical activity (i.e. work including much heavy lifting, carrying, pushing and standing) have an increased risk of cardiovascular disease and premature mortality [11], [13]. This statement is particularly relevant for individuals exposed to physically demanding work who also have a low cardio-respiratory fitness [14]. These findings suggest that persons with physically demanding work may have the same need and benefit from leisure time physical activity as persons with sedentary work.

Because of the negative cardiovascular and metabolic effects of excessive sedentary time per day [1]–[3], leisure time physical activity may be hypothesized to impose a larger preventive effect on cardiovascular disease and mortality on sedentary workers than persons with high occupational physical activity. However, this assumption has to our knowledge not previously been verified in large prospective cohort studies with repetitive measures on physical activity among both sex.

Accordingly, we investigated the preventive effect and survival benefit of leisure time physical activity on cardiovascular and all-cause mortality in the large Danish prospective cardiovascular epidemiological study – the Copenhagen City Heart Study.

## Methods

### Study design and population

The Copenhagen City Heart Study is a prospective population study in which a random sample of the population living in an area of Copenhagen is invited to participate at regular intervals. Details of the enrolment and examination are described elsewhere [15].

**Figure 1:**
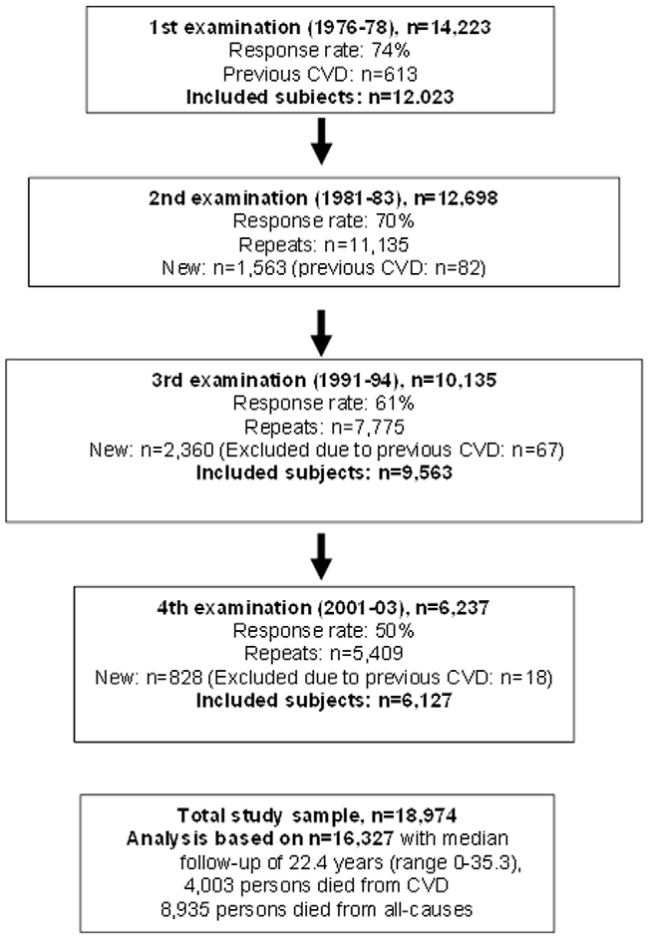
Flow diagram of the study population in the Copenhagen City Heart Study. The entire study sample consisted of persons participating in at least one of the four examinations (i.e. some persons participated in multiple examinations) in the Copenhagen City Heart Study who were free of previous cardiovascular disease (CVD) at their first examination in the study.

In short, 14,223 persons (response rate 74%) participated in the first examination in 1976–78. In 1981–83, 1991–94, and 2001–03, these participants were re-examined and new, primarily young subjects were enrolled (see Figure 1 for flowchart). A total of 18,974 subjects participated in one or more of the examinations. Participants with previous myocardial infarction or stroke by self-report or according to the Danish National Patient Register established in 1977 until study inclusion were excluded. Moreover, all persons above the age of retirement in Denmark at the time of study inclusion (i.e. 67 years of age) were excluded, leaving 16,327 persons eligible for analyses. The participants with missing observations for physical activity at work at all examinations were excluded (n = 739).

The median duration of follow-up was 22.4 years (range 0.01–35.3). Cardiovascular risk factors were assessed at each of the four examinations using the same standardised and validated methods as previously described in detail [15]. Data were obtained from a self-administered questionnaire, a physical examination, and clinical tests.

### Ethical approval

The Committee of Biomedical Research Ethics for the Capital region in Denmark approved the study (H-KF-01-144/01). All data was de-identified and analyzed anonymously. The participants provided written consent to participate in the study. This consent procedure was approved by the ethics committee.

### Predictive variables

A single question with four answer options was applied for measuring occupational physical activity: ‘Which description most precisely covers your pattern of physical activity at work? [16].

1. You are mainly sedentary and do not walk much around at your workplace. *E.g*. desk work, work including assembling of minor parts. [Score 1].
2. You walk around quite a bit at your workplace but do not have to carry heavy items. *E.g*. light industrial work, non-sedentary office work, inspection and the like. [Score 2].
3. Most of the time you walk, and you often have to walk up stairs and lift various items. Examples include mail delivery and construction work. [Score 3].
4. You have heavy physical work. You carry heavy burdens and carry out physically strenuous work. *E.g*. work including digging and shoveling. [Score 4]’.

Because of very few females in the highest category of occupational physical activity, the variable was categorised into: score 1 =  “low”, score 2 =  “moderate”, and score 3–4 =  “high”. For males, the four categories of occupational physical activity were applied and termed: score 1 =  “low”, score 2 =  “moderate”, score 3 =  “high”, and score 4 =  “very high”.

A single question with four answer options was applied for measuring leisure time physical activity:

‘Which description most precisely covers your pattern of physical activity during leisure time? [16].

1. Being almost entirely sedentary (e.g., reading, watching television or movies, engaging in light physical activity such as walking or biking for less than 2 hours per week). [Score 1].
2. Engaging in light physical activity for 2–4 hours per week. [Score 2].
3. Engaging in light physical activity for more than 4 hours per week or more vigorous activity for 2–4 hours per week (e.g., brisk walking, fast biking, heavy gardening, sports that cause perspiration or exhaustion). [Score 3].
4. Engaging in highly vigorous physical activity for more than 4 hours per week or regular heavy exercise or competitive sports several times per week. [Score 4]’.

Because of very few females and males in the highest category of leisure time physical activity, the variable was categorised into: score 1 =  “low”, score 2 =  “moderate”, and score 3–4 =  “high”.

### Covariates

Potentially confounding factors for the association between occupational and leisure time physical activity and cardiovascular and all-cause mortality were measured as follows:

Information on smoking habits was self-reported based on a single-item question, and the study participants were categorized as never smokers, ex-smokers, and current smokers of 1–14, and ≥15 cigarettes per day.

Information on alcohol consumption was self-reported, and the study participants were categorized in the statistical analyses as abstainers, or monthly, weekly, or daily consumers.

Household income was self-reported based on average income per month within the last year and categorized as low, medium, and high.

Diabetes was self-reported or a non-fasting blood glucose ≥11.1. Treatment for hypertension was self-reported, and categorized as yes/no.

Systolic blood pressure was measured in a sitting position after 5 minutes of rest, and applied as a continuous variable in the statistical analysis.

Body mass index (BMI) was calculated as measured weight (kg) divided by measured height squared (m^2^), and categorized for the statistical analyses as underweight (<18.5), normal weight (18.5–24.9), overweight (25–29.9) and obese (> = 30). Cholesterol was measured non-fasting in millmoles per litre.

### Follow-up

Follow-up was carried out by data linkage to national registers. Deaths were obtained until June 2011 from The Civil Registration System and causes of death from The National Register of Causes of Death until January 2010. Cardiovascular death was defined as ICD-8: 390–458 and ICD-10: I00–I99.

### Analyses

For the univariate analyses of demographics, lifestyle, and clinical factors (Table 1), Fisher's exact test was used for categorical covariates and ANOVA for continuous covariates.

**Table 1:** Demographics, lifestyle, and clinical factors of males and females between 20 and 67 years of age without history of cardiovascular disorders according to level of occupational physical activity at baseline in the Copenhagen City Heart Study.

Sex	Males					Females			
Occupational physical activity	Low n = 2,146	Moderate n = 1,911	High n = 1,738	Very High n = 905		Low n = 2,309	Moderate n = 3,473	High n = 2,316	
Age, mean (SD)	44.8 (12.7)	47.6 (11.4)	47.4 (11.1)	46.7 (10.8)	<0.001	45.2 (12.5)	49.5 (11.0)	44.1 (11.1)	<0.001
BMI, mean (SD)	25.0 (3.6)	25.4 (3.6)	25.7 (3.6)	26.6 (3.9)	<0.001	23.7 (4.1)	24.4 (4.3)	24.2 (4.3)	<0.001
Current smokers, %	60.0	66.6	69.9	73.2	<0.001	56.8	55.5	61.6	<0.001
Consuming ≥1 unit alcohol a day, %	29.8	34.8	41.6	53.2	<0.001	11.5	11.1	9.3	0.026
Low leisure time physical activity, %	17.4	13.8	15.2	19.9	<0.001	18.2	15.9	12.5	<0.001
Cholesterol, mean (SD)	5.7 (1.2)	5.8 (1.2)	5.8 (1.2)	5.9 (1.2)	<0.001	5.8 (1.4)	6.1 (1.3)	5.8 (1.2)	<0.001
Systolic blood pressure, mean (SD)	135.0 (18.8)	136.7 (19.3)	135.8 (18.8)	136.2 (17.6)	0.030	127.6 (20.1)	131.6 (21.0)	126.2 (19.1)	<0.001
Blood pressure medication, %	3.9	3.6	3.1	2.4	0.172	4.3	5.2	3.3	0.002
Diabetes, %	2.6	2.8	2.7	3.2	0.837	1.2	1.5	1.3	0.513
High household income, %	38.9	29.3	17.1	15.2	<0.001	26.0	23.4	21.8	0.005
<8 years of school education, %	18.8	33.1	47.4	58.2	<0.001	20.9	41.9	41.5	<0.001

With sex-specific multi-adjusted Cox proportional hazards regression models with time-dependent covariates and age as the underlying time scale and delayed entry (optimizing adjustment for age), the associations between leisure time physical activity and the outcomes were studied within each category of occupational physical activity. The predictive variables and covariates were attained from the examination of entry of each participant, and thereafter updated at each of the following examinations. All adjusted models included the covariates age, calendar time, smoking, alcohol consumption, BMI, occupational or leisure time physical activity, systolic blood pressure, diabetes, blood pressure medication, and household income.

Moreover, adjusted sex-specific Cox proportional hazards regression analyses were performed with a multiplicative interaction term between occupational and leisure time activity. Further, the survival benefit was calculated by integrating the survival function estimated in the Cox models. The survival benefit was calculated for a healthy average person (mean value of blood pressure, cholesterol, BMI, household income, and a never smoker, without diabetes and not taking blood pressure medication, and consuming alcohol on a monthly basis).

The assumption of proportionality in the Cox regression models was tested with the Lin, Wei, and Ying score process test [17]. Misspecifications of the functional form of the covariates were tested by plotting the continuous covariates against the cumulative residuals and compare them to random realizations under the model.

P-values below 0.05 were considered statistically significant. Statistical analyses were performed with R version 2.13.1.

## Results

During a median follow-up of 22.4 years (range 0.01–35.3), 4,003 (males: 2,087) died from cardiovascular disease and 8,935 (males: 4,401) from all-causes.

Table 1 shows demographic, lifestyle, and clinical factors according to level of occupational physical activity among males and females. Several significant differences were found. Men with very high occupational physical activity were more frequently current smokers, consumed more than 1 unit alcohol a day, had less than 8 years of school education, had a lower household income, had a higher BMI and fewer were taking blood pressure medication compared to men with low occupational physical activity.

Among females, those with a high occupational physical activity level were more frequently current smokers, were less physically active in their leisure time and more often had less than 8 years of school education compared to females with a low occupational physical activity level.

**Figure 2:**
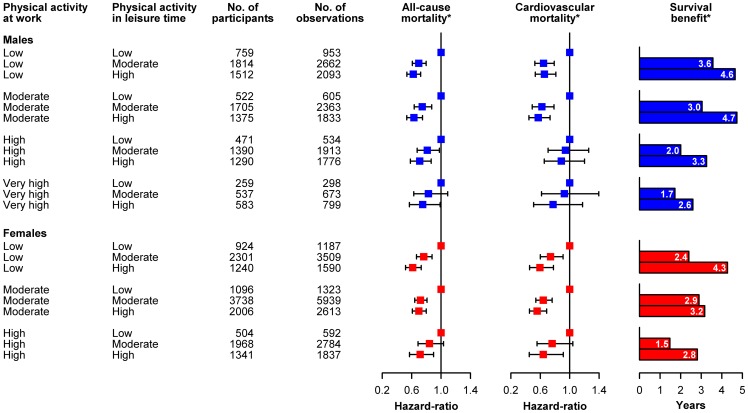
Risk (Cox regression Hazard ration with 95% confidence intervals) for cardiovascular mortality and all-cause mortality from leisure time physical activity adjusted for age, calendar time, BMI, smoking, alcohol consumption, cholesterol, systolic blood pressure, blood pressure medication, diabetes and household income stratified on occupational physical activity among males (n = 7,411) and females (n = 8,916) between 20 and 67 years of age without a history of cardiovascular disorders in the Copenhagen City Heart Study. * Adjusted for age, calendar time, BMI, smoking, alcohol consumption, cholesterol, systolic blood pressure, blood pressure medication, diabetes and household income, survival benefit was calculated for a healthy average person (mean value of BMI, systolic blood pressure, cholesterol, household income, and a never smoker, without diabetes and not taking blood pressure medication, and consuming alcohol on a monthly basis) by integrating the survival function estimated in the Cox models.

Figure 2 illustrates the risk estimates for all-cause mortality and cardiovascular mortality from leisure time physical activity within each strata of occupational physical activity among males and females adjusted for age, calendar time, BMI, smoking, alcohol consumption, cholesterol, systolic blood pressure, blood pressure medication, diabetes and household income. As seen in the figure, irrespective of occupational physical activity level, a consistently lower risk with increased leisure time physical activity was found for both cardiovascular disease and all-cause mortality. This was true for both men and women. However, with respect to cardiovascular disease mortality, the inverse association seemed to be less pronounced and did not reach statistical significance among men with high and very high occupational physical activity. To examine this closer, we performed an additional multi-adjusted model investigating the risk for all-cause and cardiovascular disease mortality with an interaction term (table 2). Corresponding with the consistent risk reductions from higher levels of leisure time physical activity in all groups of occupational physical activity (table 2), no statistical interaction between leisure time physical activity and occupational physical activity in either men or women for cardiovascular mortality (P>0.14) and all-cause mortality (P>0.47) were found. Hence, the effect of leisure time physical activity on cardiovascular and all-cause mortality seems to be independent of the level of occupational physical activity.

**Table 2:** Risk (Cox regression hazard ratios with 95% confidence intervals) for cardiovascular mortality and all-cause mortality from the interplay between occupational and leisure time physical activity adjusted for age, calendar time, BMI, smoking, alcohol consumption, cholesterol, systolic blood pressure, blood pressure medication, diabetes and household income stratified on occupational physical activity among males (n = 7,411) and females (n = 8,916) between 20 and 67 years of age without a history of cardiovascular disorders in the Copenhagen City Heart Study.

Physical activity at work	Leisure time physical activity	All-cause mortality				Cardiovascular mortality			
		Cases	HR	95% CI	p-value	Cases	HR	95% CI	p-value
**Males**									
Low	Low	348	1.64	1.41, 1.89	p<0.001	182	1.54	1.25, 1.88	p<0.001
Low	Moderate	656	1.13	1.00-1.28	p = 0.045	311	0.98	0.83, 1.16	p = 0.838
Low	High	433	1.00	Reference		236	1.00	Reference	
Moderate	Low	215	1.60	1.36, 1.89	p<0.001	114	1.59	1.25, 2.01	p<0.001
Moderate	Moderate	603	1.19	1.05, 1.35	p = 0.006	266	0.98	0.82, 1.17	p = 0.833
Moderate	High	379	1.01	0.88, 1.16	p = 0.879	181	0.91	0.75, 1.11	p = 0.340
High	Low	166	1.48	1.22, 1.79	p<0.001	70	1.15	0.87, 1.52	p = 0.317
High	Moderate	483	1.21	1.06, 1.38	p = 0.004	221	1.07	0.89, 1.28	p = 0.500
High	High	373	1.07	0.93, 1.23	p = 0.334	184	1.01	0.83, 1.23	p = 0.903
Very high	Low	84	1.65	1.30, 2.09	p<0.001	36	1.36	0.95, 1.95	p = 0.093
Very high	Moderate	161	1.33	1.10, 1.62	p = 0.004	76	1.24	0.95, 1.63	p = 0.110
Very high	High	160	1.20	1.00, 1.45	p = 0.055	68	1.01	0.77, 1.32	p = 0.938
**Females**									
Low	Low	386	1.58	1.34, 1.87	p<0.001	181	1.59	1.22, 2.07	p<0.001
Low	Moderate	699	1.22	1.05, 1.41	p = 0.007	293	1.20	0.95, 1.51	p = 0.133
Low	High	219	1.00	Reference		94	1.00	Reference	
Moderate	Low	448	1.51	1.29, 1.78	p<0.001	217	1.63	1.27, 2.10	p<0.001
Moderate	Moderate	1295	1.08	0.94, 1.24	p = 0.268	551	1.03	0.83, 1.29	p = 0.781
Moderate	High	492	1.04	0.89, 1.21	p = 0.642	184	0.89	0.69, 1.13	p = 0.335
High	Low	131	1.52	1.23, 1.89	p<0.001	59	1.72	1.24, 2.40	p = 0.001
High	Moderate	416	1.24	1.06, 1.46	p = 0.008	160	1.25	0.96, 1.62	p = 0.095
High	High	210	1.07	0.89, 1.29	p = 0.489	75	1.03	0.76, 1.39	p = 0.870

To further illustrate the impact of leisure time physical activity on longevity within different occupational physical activity groups, figure 2 also shows the estimated increase in life expectancy. Among the fourteen groups who had a moderate or high leisure time physical activity level, an increased life expectancy was calculated for all groups, ranging from 1.5 years to 4.7 years, and consistently being highest among those with the highest level of leisure time physical activity in all groups of occupational physical activity.

## Discussion

The results of this study support that leisure time physical activity is associated with a reduced risk of cardiovascular as well as all-cause mortality among men and women independent of their level of occupational physical activity.

Overall, moderate leisure time physical activity was associated with a reduction in risk of cardiovascular and all-cause mortality ranging from 6 to 38% depending on level of occupational physical activity. An even more pronounced reduction in risk, ranging from 11 to 44%, was found among individuals with a high leisure time physical activity level. Moreover, the increase in life expectancy for both sex ranged from 1.5–3.6 years for moderate and 2.6–4.7 years for high leisure time physical activity among the different levels of occupational physical activity.

As shown in table 2, high leisure time physical activity consistently reduced the risk for all-cause and cardiovascular mortality in all groups of occupational physical activity. This was also confirmed by the non-significant interactions between occupational physical activity, leisure time physical activity and the outcomes. Hence, the effect of leisure time physical activity on cardiovascular and all-cause mortality seems to be independent of the level of occupational physical activity.

Because of the well documented increased risk for CVD from sedentary behaviour [1]–[3], the considerable survival benefit from leisure time physical activity among persons with sedentary work are not surprising. However, for a considerable proportion of the working population, work constitutes the main domain for physical activity [11]. Therefore, workplace health promotion, public campaigns and international recommendations for increasing physical activity in the adult population mostly target persons with sedentary occupations [9]. The findings of this study demonstrate that leisure time physical activity not only ought to be recommended and promoted for employees in sedentary occupations, but also to employees in physically active occupations.

In another recent publication from the Copenhagen City Heart Study, men who were sedentary in leisure time had no benefit from being exposed to physically demanding work [4]. In contrast, they had an increased risk of myocardial infarction (fatal and non-fatal), and all-cause mortality [4]. This finding is in agreement with our previous observation in the Copenhagen Male Study, that among men with high physical work demands a particularly high risk of ischaemic heart disease and all-cause mortality was observed if they also had a low leisure time physical activity level and a low cardio-respiratory fitness [18], [19].

As shown in table 2, high levels of occupational physical activity does not seem to infer the same preventive effect on cardiovascular and all-cause mortality as high levels of leisure time physical activity. This is in accordance with previous studies not finding any protective effect from high physical work activity, but rather a risk enhancing effect on cardiovascular and all-cause mortality [11], [20]. This may be explained by the different characteristics of leisure time and occupational physical activity [21]. Leisure time physical activity is characterized by dynamic body movements of relatively short duration with high intensity and variation allowing for sufficient recovery while occupational physical activity is characterized by static body movements of long duration and low to moderate intensities which may occur without sufficient time for recovery [21]. This is supported by a recent study showing that leisure time physical activity is associated with a lowered diurnal ambulatory blood pressure while occupational physical activity is associated with an enhanced diurnal ambulatory blood pressure [22].

### Strengths and Limitations

The main strengths of the present study are the relatively long follow-up time, control for socioeconomic factors, repeated measures of exposure and risk factors, several objective measures of risk factors for cardiovascular disease, and inclusion of both males and females. A limitation of the study is lacking control for psychosocial work factors. However, previous studies have shown that control for psychosocial factors have minimal influence on the association between occupational physical activity and cardiovascular disease and mortality [10], [11]. Another limitation of the present study is that the information of occupational and leisure time physical activity was based on self-assessment, which invariably entails some degree of misclassification [23]. Moreover, the information of occupational and leisure time physical activity available in this study does not provide valid estimates of physical activity energy expenditure or related measures of total volume of physical activity.

### Conclusion

Among both men and women leisure time physical activity was inversely associated with risk of CVD mortality and all-cause mortality independent of the level of occupational physical activity. The implications of the findings in this study may be that future public campaigns and initiatives for increasing physical activity in the working population should target everybody, irrespective of their level of occupational physical activity.
